# Sedentariness: A Need for a Definition

**DOI:** 10.3389/fpubh.2018.00372

**Published:** 2018-12-21

**Authors:** Valentin Magnon, Frédéric Dutheil, Catherine Auxiette

**Affiliations:** ^1^Université Clermont Auvergne, UFR de Psychologie, Sciences Sociales, Sciences de l'Éducation, CNRS, LaPSCo, Clermont-Ferrand, France; ^2^Université Clermont Auvergne, CNRS, LaPSCo, Physiological and Psychosocial Stress, University Hospital of Clermont-Ferrand, CHU Clermont-Ferrand, Preventive and Occupational Medicine, WittyFit, Clermont-Ferrand, France; ^3^Faculty of Health, School of Exercise Science, Australian Catholic University, Melbourne, VIC, Australia; ^4^Université Clermont Auvergne, CNRS, LaPSCo, Clermont-Ferrand, France

**Keywords:** sedentariness, sedentary behavior, health, sitting, standing, physical activity

Sedentary behavior is a growing field of research which is now recognized as a public health issue (1). Sedentary comes from the latin term “sedere,” which means “to sit.” Many researchers have shown that remaining seated for prolonged periods of time can lead to several detrimental health effects at the physiological level [see (2) for a systematic review], such as an increased risk of developing cardiovascular disease (3–5), type 2 diabetes (3, 4, 6), obesity (4, 7), some cancers (8, 9), or musculoskeletal disorders (10, 11). It also has detrimental effects at the psychological level, such as depression (12–14), anxiety (14–16), or cognitive decline [(17–19); for a review, see 20]. However, there is no consensus in the physiological results (for contradictory results concerning cardiovascular disease and type 2 diabetes, see 21, 22, respectively), and psychological results are still not consistent for depression (23), anxiety (24), or cognitive decline (17). More research is therefore needed to further understand the implications of sedentary behavior on both physiological (25) and psychological health (17).

Sedentary behavior has been defined in many ways over the years, but mostly by using energy expenditure, i.e., any behavior where energy expenditure is strictly below 2 METs (Metabolic Equivalent Tasks) (26), or between 1 MET and 1.8 METs (27). However, that criterion alone is not enough to clarify what sedentary behavior is. Indeed, standing has an energy cost close to sitting (28), without causing any of the detrimental health effects of sedentary behavior. For instance, breaking up prolonged sitting times by standing for a few minutes has a positive impact on postprandial glucose metabolism (29–31). However, there is no consensus for these results (32–34). Posture therefore must be taken into account to distinguish a standing up position from sedentary behavior. Currently, the terminology consensus project from the sedentary behavior research network [SBRN, (35)] defines sedentary behavior as “*any waking behavior characterized by an energy expenditure* ≤ *1.5 METs, while in a sitting, reclining or lying posture*.” This definition excludes a standing position as sedentary behavior and grants a better understanding of what sedentary behaviors are, and what differentiates them from others.

However, this recent definition may not be enough to reveal the complexity of sedentariness as a way of life. Indeed, it refers to behaviors adopted at a specific time but without providing any long-term information. It does not grant a better understanding of what a sedentary lifestyle is and what its characteristics are. Thus, it fails to define what a sedentary lifestyle is, whereas such a definition has been given for physical (in)activity. According to the WHO recommendations (36), healthy adults “*should do at least 150 minutes of moderate-intensity aerobic physical activity throughout the week, or do at least 75 minutes of vigorous-intensity aerobic physical activity throughout the week, or an equivalent combination of moderate-and vigorous-intensity activity. Aerobic activity should be performed in bouts of at least 10 minutes duration*.” Yet around one third of the world's population (37) and 55 to 70% of the US population over 65 (38) do not meet these guidelines, which is detrimental to health. For example, in the general population, physical inactivity is independently related to an increased risk of obesity (39), type 2 diabetes (40, 41), some cancers (42, 43), shortened life expectancy (42, 44), and risks of mental health problems (45, 46). In Canada, physical inactivity represents nearly 3% of the overall health care costs (47) and has been recognized as a worldwide economic burden (48). These cut-offs in physical activity allow a distinction to be made between physically active individuals and physically inactive ones. They would also be useful in the definition of sedentary behaviors, but they have not yet been integrated.

Only taking into account the energy expenditure and the posture is not enough when one is interested in the lifestyle of an individual. A sedentary lifestyle cannot be reduced to physical inactivity. Regardless of the confusion between the two terms (4, 49, 50), physical inactivity and sedentary behavior can be considered as independent, because their physiological consequences on health are not the same. For example, sedentary behavior leads to an increased risk of mortality (1, 51–53), irrespective of the amount of physical activity practiced. A person can meet the guidelines regarding physical activity and still be considered as sedentary if they spend a large amount of their day sitting or lying down at home, at school, at work, driving, or during their leisure time. It is therefore important to distinguish physical inactivity from sedentariness.

Defining what sedentariness is as a lifestyle would be complementary to the definition of sedentary behavior at any given moment. Furthermore, defining sedentariness as a lifestyle is an essential step in proposing recommendations when they are needed (as for physical activity), which could have a positive impact on the quality of life (i.e., physical, psychological, and social health and, hence, the overall health of an individual) (54). However, studying sedentariness is complex since several factors may influence the relationship between sedentary behavior and mental or physical health, such as the *type of sedentary activities*. For instance, strong evidence has been found regarding the association between cardiovascular diseases and TV viewing, total sitting time and screen time. However, evidence is weak for occupational sitting (25). Regarding psychological outcomes, cognitively demanding activities (e.g., sitting while reading, actively using a computer) are associated with better executive performances such as working memory (55, 56), mental flexibility (56) and visual spatial memory (55), while TV viewing is negatively associated with such executive performances among adults (55, 56). Moreover, physical activity and sedentariness might be two factors influencing each other in the long term (57–59). High levels of moderately intensive physical activity might attenuate the increased risk of death associated with high sitting times (57). Thus, when investigating sedentariness, the *level of physical activity* might also be considered. The same applies for *age*, since older individuals have been sedentary for longer periods of time than younger ones (60). A fourth factor to consider is the *interruption of sedentary bouts*. Health benefits have been demonstrated by breaking sedentary behavior every 20 (61, 62) or 30 min (6, 30, 63, 64). Yet, there is no consensus on the preferential frequency for breaking up sedentary time. Therefore, more studies are needed in order to gain a better understanding of the full scope of the consequences of sedentary behavior on health and at which point those consequences might be detrimental in the long term.

As mentioned earlier, thresholds would be useful in the definition of sedentariness as a lifestyle as it would allow sedentary individuals to be distinguished from non-sedentary ones. According to the Australian government (65), the cut-off point for mortality risk is approximately 7 or 8 h a day. Likewise, a meta-analysis on 6 studies among adults reported that sitting for more than 7 h a day is associated with an increased mortality risk (66). However, this meta-analysis disagrees with another which was (57) conducted on 13 studies and which revealed that sitting for more than 4 h a day is enough to increase the risk of all-cause mortality among adults. This inconsistency could be explained by the way in which sedentary behaviors are evaluated, as the evaluations were objective in some cases (e.g., with accelerometers), but subjective in others (questionnaires). However, questionnaires tend to underestimate total sedentary time (67, 68) compared to objective measures (18). This hypothesis was supported by a meta-regression analysis (69) which showed that the cut-off of daily sedentary time for self-reporting studies was 7 h/day compared with 9 h/day for device-based ones.

This lack of consensus has led researchers to use different criteria to define a sedentary lifestyle, making comparisons of the results difficult. A common definition of sedentariness would improve its study and the determination of the potentially detrimental consequences on the physiology, as well as other aspects that would impact the quality of life of individuals and their well-being. For example, sitting for less than 8 h a day is associated with higher perceived mental health and vitality scores (54) and lower anxiety (16, 54) and depression scores (13, 54).

Another example is the potential relationship between sedentary behavior and the increased risk of dementia (70), which currently lacks sufficient evidence (17). Recommendations for a sedentary lifestyle would enable the relationship between sitting time and an increased risk of dementia to be studied. If sedentariness is a risk factor for cognitive decline (18) and dementia (17, 70), then guidelines for sedentariness would grant the possibility of developing efficient intervention protocols in order to decrease sedentariness and make modern lifestyles healthier. Such interventions would reduce the high prevalence of dementia (71) and its economic cost (72). Therefore, a definition of sedentariness would provide a better understanding of this lifestyle and its physiological, psychological, economic and social implications. This definition should at least take into account the type of current and past sedentary activities (cognitively demanding vs. undemanding activities) and their duration, the type of current and past physical activity (low, moderate, vigorous), the way sedentarity is measured (objective measurements as far as possible), the interruption of sedentary bouts, and age.

## Author Contributions

VM reviewed the literature, created, and maintained a Zotero database, wrote the initial manuscript, and revised the second drafts following feedback from CA and FD. CA reviewed the literature. Both CA and FD made editorial suggestions for the second drafts. All authors contributed to manuscript revision, read and approved the submitted version.

### Conflict of Interest Statement

The authors declare that the research was conducted in the absence of any commercial or financial relationships that could be construed as a potential conflict of interest.
